# Prognostic Significance of Amino Acid Transporters in Endometrial Cancer

**DOI:** 10.3390/cancers18203404

**Published:** 2026-10-21

**Authors:** Mikołaj Piotr Zaborowski, Łukasz Ciecierski, Piotr Tyburski, Magdalena Nadolna, Marta Brygida Szczygieł, Jędrzej Sikora, Mateusz Szot, Stefan Malewski, Marcin Iwanicki, Kinga Piątek-Bajan, Jarosław Lewandowski, Cezary Miedziarek, Michał Potograbski, Nikodem Horst, Ewa Nowak-Markwitz

**Affiliations:** 1 Department of Gynecologic Oncology, Chair of Gynecology, Poznań University of Medical Sciences, 33 Polna Street, 60-535 Poznań, Poland; mzaborowski@ump.edu.pl (M.P.Z.); piotrtyburski00@gmail.com (P.T.); magdalena.nadolna2001@gmail.com (M.N.); jedrzejsikora@gmail.com (J.S.); mateo.szot@gmail.com (M.S.); malewskistefan@gmail.com (S.M.); cezary.miedziarek@gmail.com (C.M.); m.potograbski@gmail.com (M.P.); 2 Institute of Bioorganic Chemistry, Polish Academy of Sciences, Noskowskiego 12/14, 61-704 Poznań, Poland; lciecierski@ibch.poznan.pl (Ł.C.); kbajan@ibch.poznan.pl (K.P.-B.); jarekl@man.poznan.pl (J.L.); 3 Student Scientific Society, Faculty of Medicine, Poznań University of Medical Sciences, 10 Fredry Street, 61-701 Poznań, Poland; 4 National Medical Institute of the Ministry of the Interior and Administration, 02-507 Warsaw, Poland; 5 Department of Chemistry and Chemical Biology, Stevens Institute of Technology, Hoboken, NJ 07030, USA; miwanick@stevens.edu; 6 Department of General and Colorectal Surgery, Poznan University of Medical Sciences, 10 Fredry Street, 61-701 Poznań, Poland; nvhorst@gmail.com

**Keywords:** endometrial carcinoma, solute carrier transporters, the cancer genome atlas, mRNA expression, SLC38A1, SLC38A3, SFXN1, SLC1A4, survival analysis, molecular subtypes

## Abstract

**Background:** Identification of high-risk patients with endometrial cancer (EC) remains challenging despite advances in molecular profiling. **Methods:** We comprehensively analyzed the RNA expression of 18 amino acid transporters (AATs) in 373 EC samples from The Cancer Genome Atlas (TCGA). **Results:** Our clustering analysis based on AAT expression identified a patient group with a significantly worse prognosis. Sideroflexin family (SFXN1, SFXN5), glutamine (SLC38A1, SLC38A3), glycine (SLC6A9) transporters, and the heavy-chain subunit of heteromeric AATs (SLC3A2) were elevated in poorly differentiated tumors. In contrast, the transporter of small neutral amino acids (SLC1A4) and proline/imino-acid transporter (SLC6A20) were enriched in lower-grade disease. SFXN1 and SLC38A1 were also higher in advanced (III/IV) FIGO stages. AAT expression differed across molecular subtypes, with SFXN5, SLC38A1, SLC38A3, and SLC3A2 associated with the copy-number-high profile. In survival analysis, we demonstrated that SLC38A3, SFXN4, and SLC3A2 were associated with shorter disease-free survival, and SLC38A1, SLC38A3, and SFXN1 with worse overall survival, whereas SLC1A4, SLC6A20, and the taurine transporter (SLC6A6) were related to favorable outcomes. **Conclusions:** These findings highlight AAT expression as a potential marker of EC aggressiveness and prognosis and suggest that integrating metabolic features with molecular classification may improve risk stratification and identify potential therapeutic targets.

## Simple Summary

Identifying patients with endometrial cancer who are at greater risk of disease progression or poor survival remains a challenge. Cancer cells require amino acids to grow and survive. Proteins that transport these nutrients into and within cells may play an important role in tumor development. In this study, we investigated the expression of 18 amino acid transporters in tumor samples from 373 patients with endometrial cancer to determine whether their expression patterns are associated with tumor aggressiveness and patient outcomes. We identified distinct groups of patients based on transporter expression, including one with significantly poorer survival. Several transporters were associated with more aggressive tumor characteristics and unfavorable outcomes, while others were linked to better prognosis. Our findings suggest that amino acid transporters could serve as potential biomarkers to improve risk assessment and may provide new insights into metabolic vulnerabilities that could be explored as therapeutic targets.

## 1. Introduction

Endometrial cancer (EC) is the most prevalent gynecological cancer worldwide, with over 400,000 new cases and 100,000 deaths reported annually [1,2]. While localized EC has a favorable five-year overall survival (OS) rate of 95%, the prognosis is markedly worse for advanced disease, with the OS rate of 19% [3]. Precisely identifying patients who require intensified therapy versus those suitable for treatment de-escalation remains a significant clinical challenge.

Over the past decade, the TCGA analysis has revolutionized EC classification by identifying four prognostic molecular subtypes that stratify patients by recurrence risk: POLE-mutant (POLEmut; also known as ultramutated, excellent prognosis), mismatch repair-deficient (MMRd; known as hypermutated, intermediate prognosis), non-specific molecular profile (NSMP; known as copy-number-low, intermediate prognosis), and p53-abnormal (p53abn; known as copy-number-high, poor prognosis). In the current clinically applicable molecular classification of endometrial cancer, the TCGA-defined copy-number-high and copy-number-low subgroups correspond approximately to the p53-abnormal (p53abn) and no specific molecular profile (NSMP) subtypes, respectively. However, these classifications are not strictly interchangeable [4]. The latest 2025 ESGO/ESTRO/ESP guidelines recommend molecular profiling to achieve precise risk stratification and appropriate treatment. They indicated an additional subgroup of estrogen receptor-negative tumors with an unfavorable prognosis [5]. The PORTEC-3 and PORTEC-4a trials also highlighted that integrating molecular data should guide adjuvant treatment, allowing de-escalation in favorable-risk groups (e.g., POLE-mutated) while intensifying therapy for high-risk profiles (e.g., copy-number-high) [6]. Furthermore, trials such as RUBY and DUO-E have demonstrated substantial survival benefit from adding immune checkpoint inhibitors to chemotherapy in specific molecular subgroups [7,8]. Ultimately, the modern treatment of EC is strictly personalized based on distinct molecular profiles. Despite these advancements in molecular profiling, a proportion of patients are still misclassified according to current criteria. Consequently, some patients receive insufficient treatment, increasing their risk of relapse, while others are overtreated and suffer from associated toxicities. From a clinical perspective, discovering novel biomarkers and integrating them with established clinico-molecular features are important for refining patient stratification into low- and high-risk groups and ultimately improving therapeutic outcomes [5].

Amino acid transporters (AATs) are integral membrane proteins located at the plasma membrane or within intracellular membranes (e.g., mitochondria and lysosomes). They are essential for tumor nutrient acquisition and are considered a potential therapeutic target [9,10]. Cellular proliferation and survival in malignant tumors depend on a continuous supply of amino acids, which serve as essential components of protein synthesis and as vital carbon and nitrogen donors for nucleotide production [9,11]. Cancer cells, including EC cells, systematically change their amino acid metabolism to bypass traditional checkpoints, directly fueling the tricarboxylic acid cycle [12,13]. Since these processes are critical for the development of EC, understanding these cellular mechanisms, including those involving AATs, is beneficial for determining potential therapeutic targets [9]. Humans possess numerous AATs, which tumor cells regulate to meet their metabolic demands [14]. Several AATs have already been established as promising footholds for advanced treatment of various cancers [15,16,17]. Identifying the specific AATs used by EC cells could enable better evaluation of recurrence risk and, potentially, the development of a new class of drugs designed to deprive tumor cells of crucial nutrients for patients with advanced or recurrent EC. Therefore, this study aimed to analyze the TCGA database to investigate the relationships between AAT expression profiles and the clinical and molecular characteristics of EC.

## 2. Materials and Methods

### 2.1. Data Retrieval

We used the UniProt database to identify AAT markers by reviewing their functions. Ultimately, 18 markers with complete transcriptome data in TCGA were selected: SLC3A2, SLC6A6, SLC1A4, SLC6A9, SLC7A5, SFXN4, SLC66A1, SLC15A3, SLC15A4, SFXN5, SLC25A38, SFXN2, SLC38A3, SLC6A7, SFXN3, SLC38A1, SFXN1, SLC6A20 (Table 1). Transcriptome data were obtained from TCGA via http://www.cbioportal.org/api/v2/api-docs (accessed on 5 February 2024) as a cBioPortalData::cBioPortal object (cbio) compatible with downstream analysis in R (cBioPortalData package version 2.22.1). For analysis of RNA-Seq data from EC tumors, values provided in the genetic profile “ucec_tcga_pub_rna_seq_v2_mrna_median_all_sample_Zscores” in the study labeled as “ucec_tcga_pub” were selected. We used the clinical characterization of the patients obtained by the function cBioPortalData::clinicalData. All data processing was performed in the R programming language (version 4.5.2) using RStudio (version 2024.12.0 + 467 “Kousa Dogwood” Release).

### 2.2. Heatmap

The heatmap figure was prepared using the ComplexHeatmap::pheatmap function (ComplexHeatmap package version 2.26.0). Clinical features’ distributions and clustering labels overlay were created using the ComplexHeatmap::HeatmapAnnotation function. Patients described by the heatmaps’ columns were sorted by their assigned clustering label, and the intra-group dissimilarity value was calculated between each cluster’s patient pairs using the PoiClaClu::PoissonDistance function (PoiClaClu package version 1.0.2.1). For the gene expression level visualization, we have used the GSEA GenePattern color palette. To visualize the qualitative and quantitative distributions of clinical features, we have used the RcolorBrewer, rcartocolor, and pal_npg color palettes.

### 2.3. Clustering Analysis

To improve the visual analysis of the dataset’s heatmap, we performed clustering analysis of patients and gene expression features. To cluster the patients solely by their AAT gene expression profiles, we first performed noise reduction with PCA using the stats::prcomp function (stats package version 4.5.2) with parameters center = FALSE, scale = FALSE, because the data had already been standardized to z-scores. With a 90% cumulative explained variance cutoff, the features were reduced to the first 14 PCA components from the 18 original features. The cumulative explained variance was analyzed using a scree plot created with the factoextra::fviz_screeplot function (factoextra package version 2.0.0). Next, we performed dimensionality reduction in the PCA embedding to 4 dimensions using the structure-preserving UMAP algorithm and the umap::umap function (umap package version 0.2.10.0), with n_components = 4 and other parameters set to their defaults. Finally, the UMAP components were used as input to the OPTICS clustering algorithm, which we ran with dbscan::optics (dbscan package version 1.2.4) and minPts = 20. We have increased the minimum points parameter beyond the typical rule-of-thumb value of twice the input data dimensionality to smooth the reachability plot and reduce spurious clusters that arise from UMAP’s nonlinearity and local-structure-preserving capabilities. By visually analyzing the reachability plot, we manually selected a 0.86 cutoff for reachability distance to balance the number of clusters and the number of unlabeled outlier observations. The reachability plot was drawn by calling the “plot” method on the dbscan::optics result. To extract the clustering labels, we used the dbscan::extractDBSCAN method on the dbscan::optics result, with the cutoff selected by the eps_cl = 0.86 parameter. The AAT gene expression features were clustered using hierarchical clustering implemented in the ComplexHeatmap::pheatmap function (ComplexHeatmap package version 2.26.0) with the parameter cluster_rows = “hierarchical_row”.

### 2.4. Stratification of Patients by Overall and Disease-Free Survival

Disease-free survival (DFS) was defined as the time from the date of primary treatment to the first documented disease recurrence. Obtained OPTICS clustering labels were used to generate Kaplan–Meier disease-free and overall survival curves for each of the clusters. The curves were generated and plotted using the survminer::ggsurvplot function (survminer package version 0.5.1). Patients with unassigned labels, considered outliers, were omitted from the analysis. To assess stratification among patient groups, we calculated and plotted the global log-rank test by setting pval = TRUE in the survminer::ggsurvplot function. To test the separability of survival curves between the most extreme strata, we calculated and plotted the pairwise log-rank test again by providing the pval = TRUE parameter to the survminer::ggsurvplot function, using right-censored survival data from only two clusters.

### 2.5. Statistical Analysis

Normality of the gene expression z-score distributions was verified using stats::shapiro.test method and by manual examination of the quantile-quantile plots (Q-Q plots) comparing the data distributions with the normal distribution. The plots were drawn using ggplot2 functions including ggplot2::stat_qq_band, ggplot2::stat_qq_line, and ggplot2::stat_qq_point (ggplot2 package version 4.0.1). Although only half of the tested features passed the Shapiro test with a significant *p*-value < 0.05, manual inspection of the Q-Q plots revealed a consistent linear mapping of the compared distributions, with exceptional outliers at the extremes of some of the distributions. Given that, we have decided to consistently apply parametric distribution comparison tests in all of our statistical analyses. For pairwise comparison of the distributions, we have applied Welch’s *t*-test using stats::t.test (stats package version 4.5.2) method with the var.equal = FALSE parameter and without considering confounders in the test formula. For multi-group (>2) comparison of the distributions, we have applied Welch’s ANOVA test using stats::oneway.test method with the var.equal = FALSE parameter. To control the number of false positives, we adjusted all distribution comparisons using stats::p.adjust function with the Holm-Bonferroni method. For Welch’s ANOVA, we defined a family of tests as all comparisons of a given categorical variable with all considered gene expression features. For pairwise Welch’s *t*-tests between distributions of gene expression at different categories of a categorical feature with levels greater than two, we have adjusted the *p*-value to the number of possible pairwise comparisons. If the given categorical feature had only two levels, we adjusted Welch’s *t*-test for the number of tests that included this feature and one of the gene expression features. To accurately visualize individual data points of distributions, we have used the ggbeeswarm::position_beeswarm function with the method = “compactswarm” parameter (ggbeeswarm package version 0.7.3).

### 2.6. Univariate Analysis of Overall and Disease-Free Survival

To perform univariate survival analysis, we have utilized two methodologies. Firstly, we have assessed the time-to-event regression performance of the AAT gene expression features and compared it with the considered clinical parameters. For this, for each gene expression or clinical feature, we have fitted a univariate Cox proportional hazards model to patients’ right-censored survival data using the survival::coxph function (survival package version), with the formula “Surv(time_to_event, status) ~ predictor” and default parameters. To make qualitative features suitable for model fitting, we one-hot encoded their levels as separate features. For each univariate model, we report the predictor’s β coefficient, the Wald test statistic and *p*-value, and the hazard ratio estimate. We plotted the models’ results using the ggstatsplot::ggcoefstats function (ggstatsplot package version 0.13.4), showing only significant predictors with Wald’s test *p*-value < 0.05. The second analysis aimed to assess the stratification capabilities of the AAT gene expression features. For each of the features, we selected an optimal threshold stratifying the patients into low- and high-marker-level strata with the maximally selected log-rank statistic calculated using the maxstat::maxstat.test function (maxstat package version 0.7.26) and run with smethod = “LogRank”, pmethod = “condMC”, B = 10000, minprop = 0.25, and maxprop = 0.75 parameters. The maximally selected log-rank statistic plot was drawn using standard ggplot2 functions (ggplot2 package version 4.0.1). The Kaplan–Meier plot for the low- and high-marker-level patient groups was generated using the survminer::ggsurvplot function (survminer package version 0.5.1). The default *p*-value produced by survminer::ggsurvplot was overwritten with the *p*-value from the maximally selected log-rank statistic, which is adjusted to account for multiple cutpoint testing.

## 3. Results

### 3.1. Study Cohort and Identification of Patient Groups

**Figure 1:**
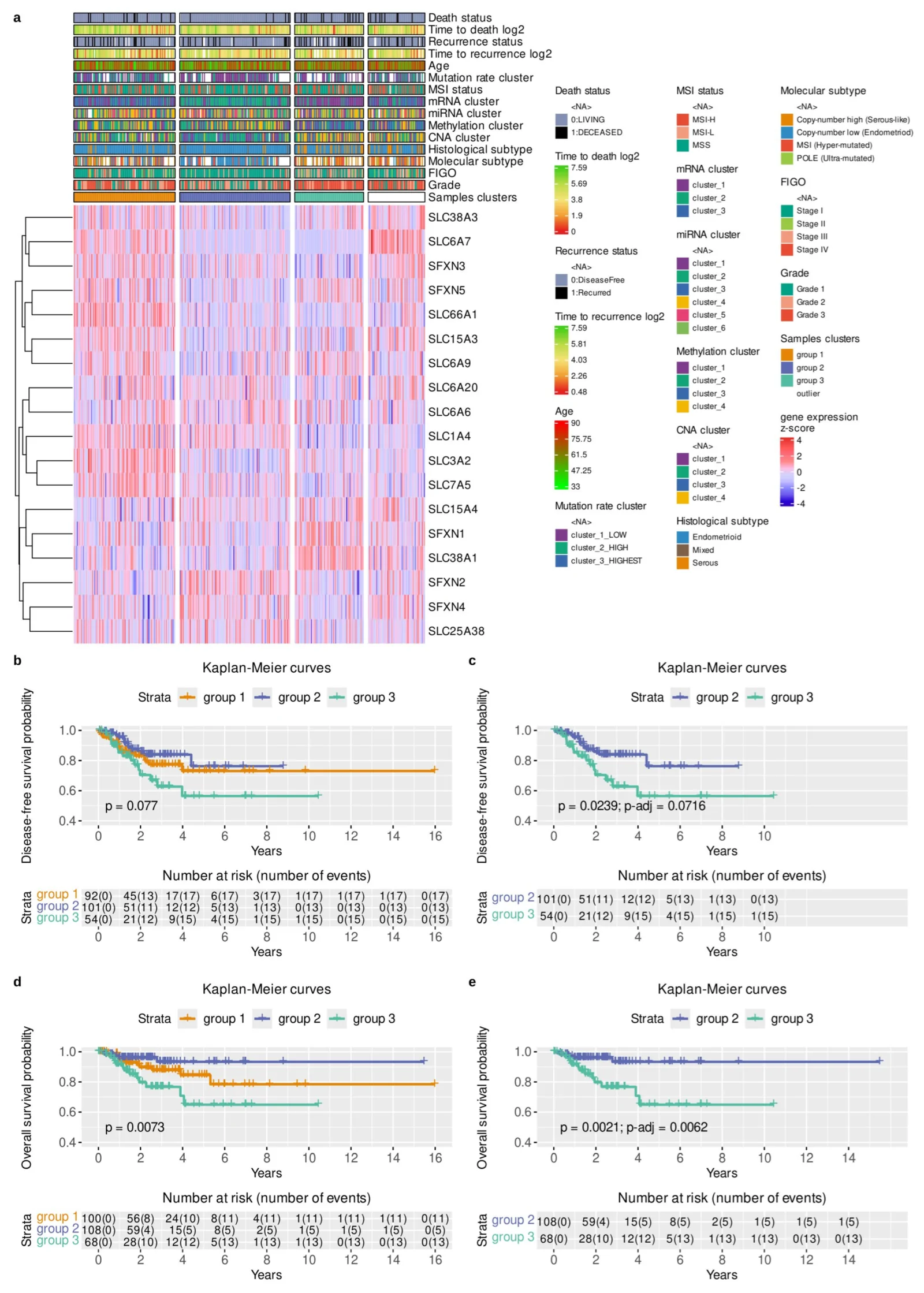
Distribution of 18 AATs in the EC population and identification of the patient group with poor prognosis. (**a**) The heatmap collectively presents the distribution of gene expression across selected clinical parameters. Patients were clustered using the OPTICS method on PCA and UMAP embedding, while genes were ordered with hierarchical clustering. Missing values for the clinical parameters and clustering outliers were marked in white. Time-to-death variable of overall survival (OS) and time-to-recurrence variable of disease-free survival (DFS), expressed in years, were winsorized at 0.2 and log2-transformed to reduce the effect of outliers and improve visualization with the color gradient. (**b**,**d**) Shows Kaplan–Meier distributions of the identified patient groups in the clustering analysis, describing DFS and OS, respectively. *p*-value expresses the results of the global log-rank test performed on all strata. Patients identified as outliers by the OPTICS method were excluded. (**c**,**e**) Shows Kaplan–Meier distributions for the two most extreme patient groups for disease-free and overall survival, respectively. To control the family-wise error rate of multiple pairwise tests, we used the Holm-Bonferroni *p*-value adjustment. The number of patients at risk at a given time and the cumulative number of events per stratum were described in the number-at-risk tables.

In this study, we used the TCGA database to analyze mRNA expression of AATs identified in the UniProt database. The cohort comprised 373 EC samples, with complete molecular data available for 18 AATs. We provide a general characterization of these AATs in Table 1. We summarized the detailed clinical, pathological, and epidemiological characteristics of the patient cohort in Table 2. While clustering patients by mRNA expression of those AATs, we noted that one of the identified groups, group 3, had a significantly worse prognosis (Figure 1a). That patient subpopulation primarily included those with a copy-number-high profile and grade 3 tumors (Figure 1a). We confirmed that patient clusters identified by AATs expression differed in DFS (Figure 1b,c) with *p* = 7.7 × 10^−2^ and in OS (Figure 1d,e) with *p* = 7.3 × 10^−3^. Specifically, we observed that ‘group 3’ had the shorter progression-free survival (*p* = 2.39 × 10^−2^, *p*-adj = 7.16 × 10^−2^, Figure 1c) and significantly shorter overall survival (*p* = 2.1 × 10^−3^, *p*-adj = 6.2 × 10^−3^, Figure 1e) than the favorable ‘group 2’. Thus, we concluded that the AATs mRNA expression profile might have prognostic relevance requiring further investigation.

### 3.2. Grade and Stage Analysis

**Figure 2:**
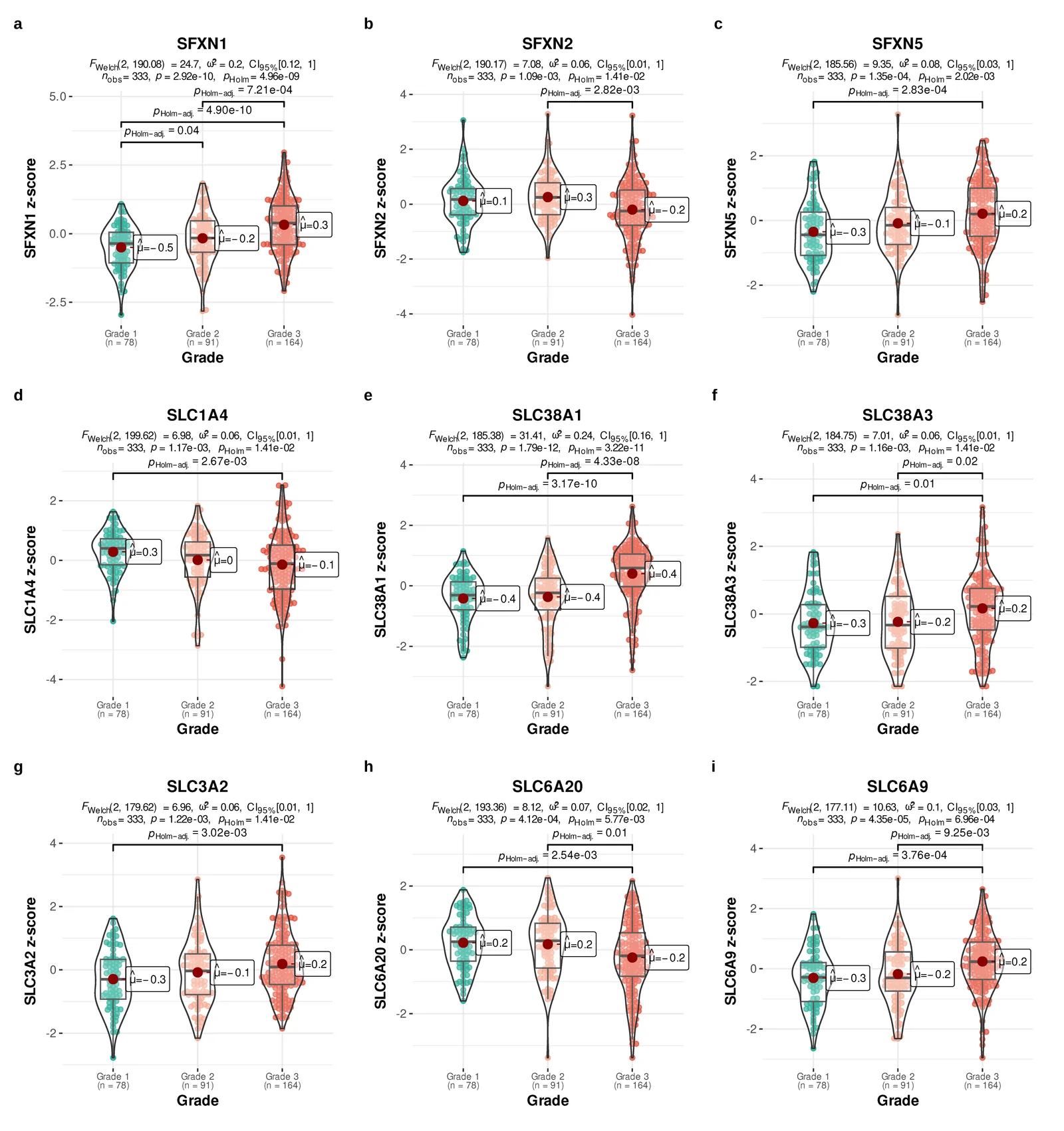
AATs expression across tumor grades (**a**–**i**). Boxplots with violin and beeswarm overlays show the distribution of gene expression z-scores across tumor grades. Multi-group comparisons were performed using Welch’s ANOVA with *p*-values adjusted for the number of tested genes using the Holm-Bonferroni method. Pairwise tests were performed using Welch’s *t*-test with its *p*-value adjusted by the number using the Holm-Bonferroni method. Only genes with significant adjusted *p*-values (*p*-adj < 0.05) from Welch’s ANOVA are shown. The number of patients (*n*) for each subgroup is stated on the plots. The median of each distribution is described by a horizontal bar, while a maroon dot indicates the mean. Labels show the rounded value of the mean.

**Figure 3:**
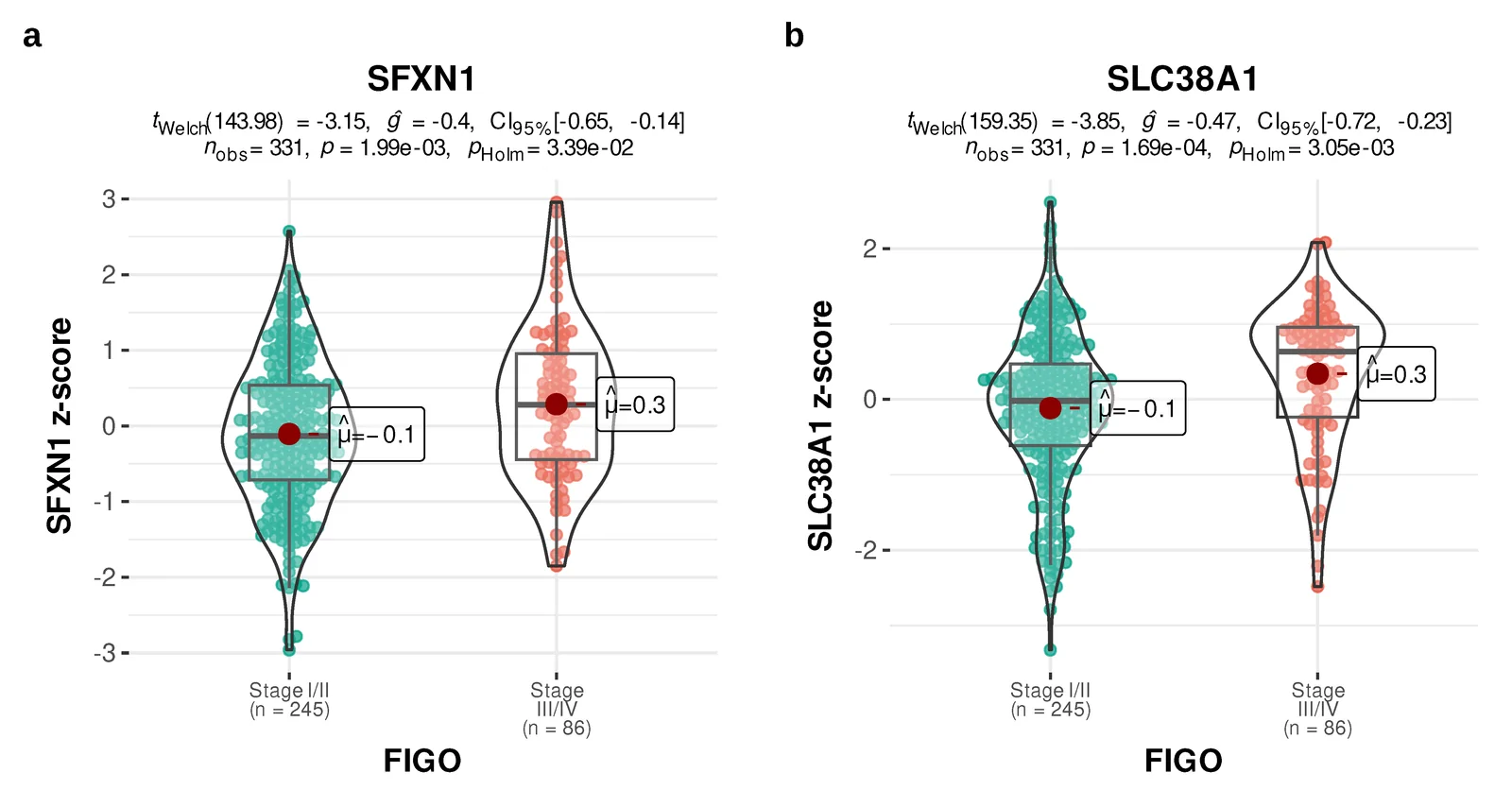
AATs’ analysis in different FIGO stages (**a**,**b**). Boxplots with violin-plot and beeswarm-plot overlays showing the distribution of gene expression z-scores across tumor stages, grouped into Stage I/II or Stage III/IV categories. Pairwise tests were performed using Welch’s *t*-test with its *p*-value adjusted by the number of tested genes with the Holm-Bonferroni method. Only genes with Welch’s *t*-test’s significant adjusted *p*-values (*p*-adj < 0.05) are shown. The number of patients (*n*) for each subgroup is stated on the plots. The median of each distribution is described by a horizontal bar, while a maroon dot indicates the mean. Labels show the rounded value of the mean.

To analyze this in detail, we evaluated AAT mRNA expression across several clinical parameters. We observed that the sideroflexin transporter family was associated with tumor differentiation. Specifically, an increasing trend in SFXN1 expression across tumor grades was observed. Its levels were significantly higher in Grade 3 compared to both Grade 1 (*p*-adj = 4.90 × 10^−10^; Figure 2a) and Grade 2 (*p*-adj = 7.21 × 10^–4^; Figure 2a). A similar pattern was observed for SFXN5 (*p*-adj = 2.83 × 10^–4^; Figure 2c), whereas SFXN2 decreased in poorly differentiated tumors (*p*-adj = 2.82 × 10^–3^; Figure 2b). Analogous profile of increasing expression values together with tumor grade were observed for glutamine transporters: SLC38A1 (Grade 3 vs. Grade 1: *p*-adj = 3.17 × 10^−10^; Grade 3 vs. Grade 2: *p*-adj = 4.33 × 10^−8^; Figure 2e) and SLC38A3 (Grade 3 vs. Grade 1: *p*-adj = 0.01; Grade 3 vs. Grade 2: *p*-adj = 0.02; Figure 2f); glycine transporter, SLC6A9 (Grade 3 vs. Grade 1: *p*-adj = 3.76 × 10^−4^, Grade 3 vs. Grade 2: *p*-adj = 9.25 × 10^−3^; Figure 2i) and the heavy-chain subunit of multiple amino acid transporters, SLC3A2 (Grade 3 vs. Grade 1: *p*-adj = 3.02 × 10^−3^; Figure 2g). Conversely, consistent with its favorable prognostic profile, neutral amino acid transporter A, SLC1A4, and transporter of glycine and proline, SLC6A20, showed higher expression in Grade 1 tumors compared to Grade 3 (*p*-adj = 2.67 × 10^−3^; Figure 2d and *p*-adj = 2.54 × 10^−3^; Figure 2h, respectively). Furthermore, the expression of two AATs was notably associated with FIGO staging (Figure 3a,b). SFXN1 expression, consistent with its relationship to grading, was clearly higher in FIGO III/IV than in FIGO I/II tumors (*p*-adj = 3.39 × 10^−2^; Figure 3a). SLC38A1 mRNA levels were also elevated in advanced FIGO stages (*p*-adj = 3.05 × 10^−3^, respectively; Figure 3b).

### 3.3. Molecular Profile Analysis

**Figure 4:**
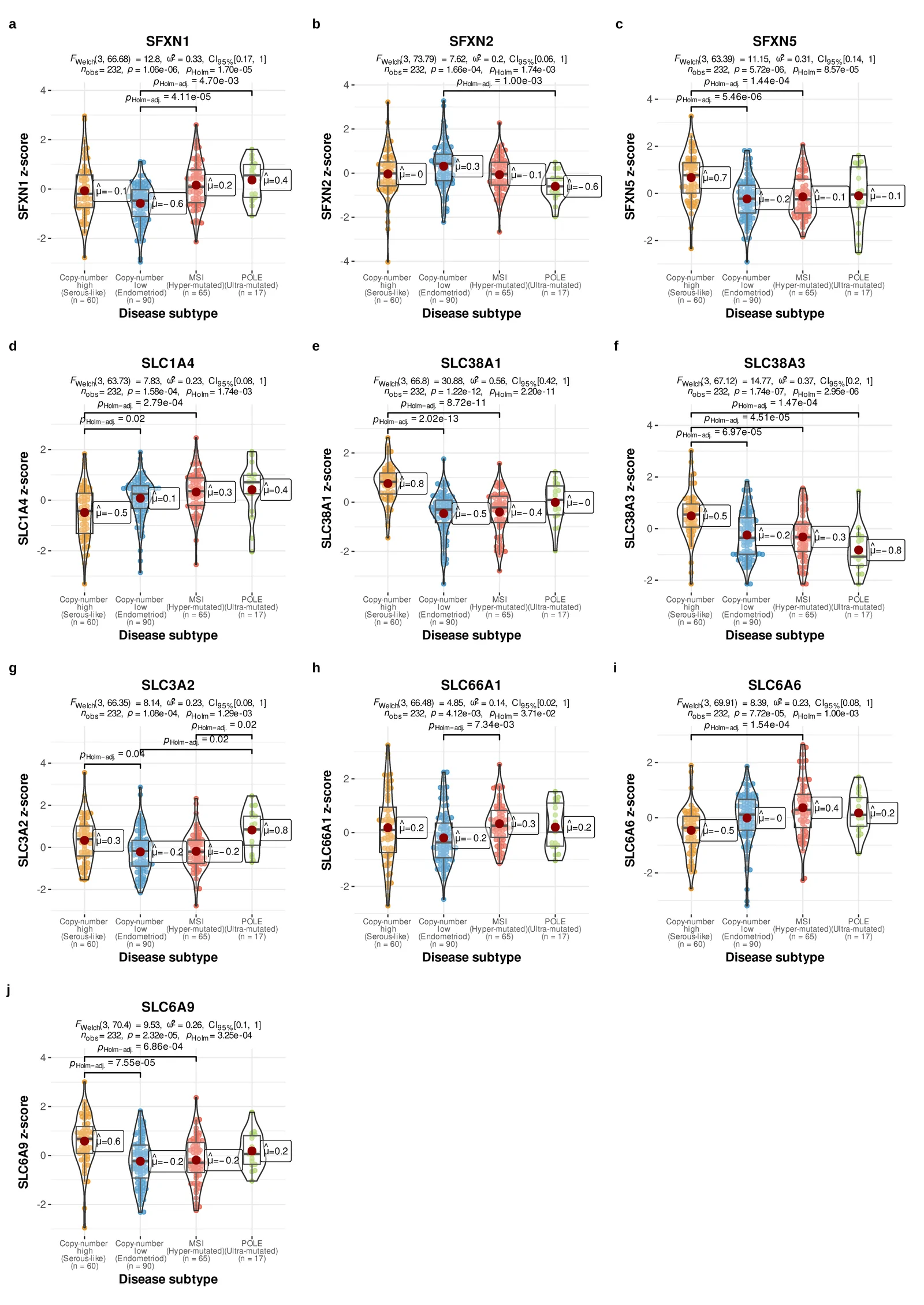
Relationship between AATs and EC molecular profiles (**a**–**j**). Boxplots with violin and beeswarm overlays showing the distribution of gene expression z-scores across disease subtypes. Multi-group comparisons were performed using Welch’s ANOVA with *p*-values adjusted for the number of tested genes using the Holm-Bonferroni method. Pairwise tests were performed using Welch’s *t*-test with its *p*-value adjusted by the number of categories with the Holm-Bonferroni method. The number of patients (*n*) for each subgroup is stated on the plots. Only genes with Welch’s ANOVA test significant adjusted *p*-values (*p*-adj < 0.05) from Welch’s ANOVA are shown. The median of each distribution is described by a horizontal bar, while the mean is indicated by a maroon dot. Labels show a rounded value of the mean.

The expression of the selected AATs was then assessed across the four TCGA molecular subtypes (Figure 4). Again, the sideroflexin transporter family differed between the subgroups. SFXN1 level was significantly lower in low-copy number as compared to other profiles (vs. POLE subgroup: *p*-adj = 4.7 × 10^−3^, vs. MSI subgroup: *p*-adj = 4.11 × 10^−5^; Figure 4a). An opposite pattern with high values in copy-number-low was demonstrated by SFXN2 (vs. POLE subgroup: *p*-adj = 1.00 × 10^−3^; Figure 4b). SFXN5 clearly increased in the copy-number-high subtype (vs. copy-number-low subgroup: *p*-adj = 5.46 × 10^−6^; Figure 4c). A similar increase in copy-number-high profile was observed for SLC38A1, SLC38A3, and SLC3A2 (*p*-adj = 2.02 × 10^−13^; *p*-adj = 6.97 × 10^−5^; *p*-adj = 0.04; as compared to copy-number-low profile, Figure 4e,f, respectively). Two transporters, SLC66A1 and SLC6A6, were more abundant in tumors with microsatellite instability (Figure 4h,i).

### 3.4. Survival Analysis

**Figure 5:**
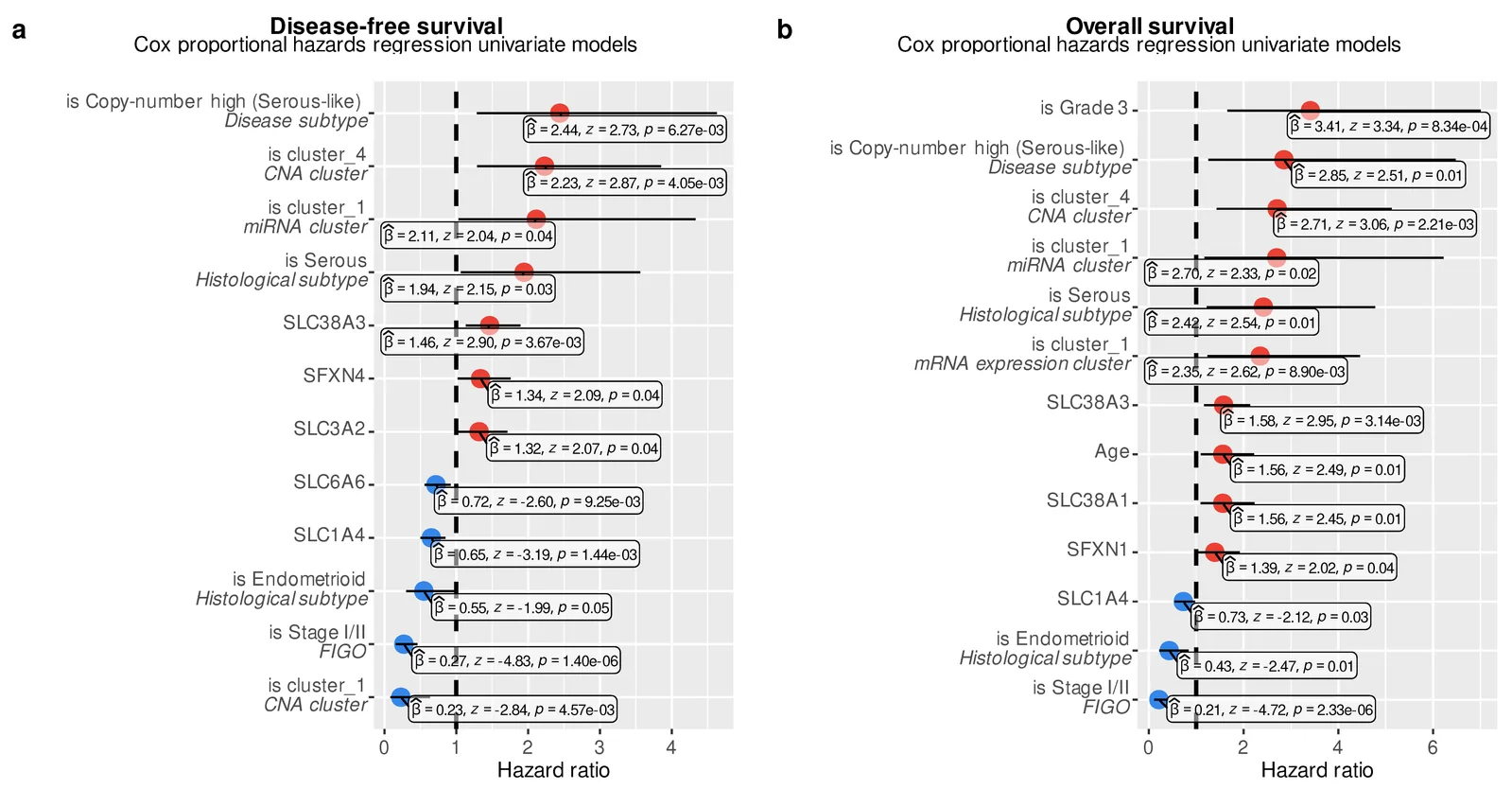
Cox regression analysis including AATs as prognostic factors. Forest plots display the results of univariate Cox regression models evaluating clinical and molecular features for disease-free (**a**) and overall survival (**b**), respectively. Categorical features labeled in cursive were one-hot encoded, with each category treated as a separate feature.

**Figure 6:**
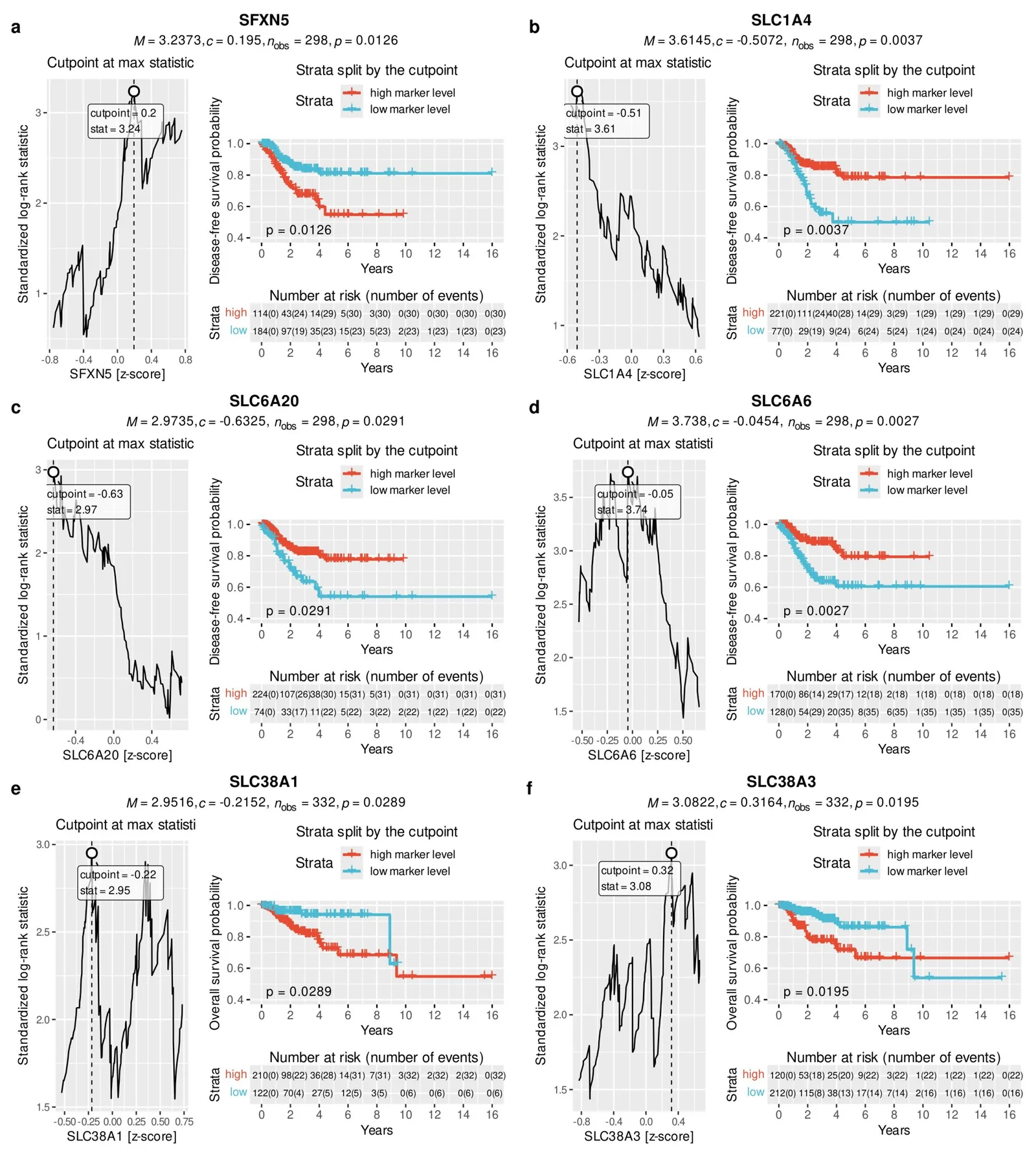
Optimization of AAT cut-off values to stratify patients into distinct survival curves (**a**–**f**). Statistically significant (*p* < 0.05) results of the separation of survival curves by an optimized cutoff value of a given gene expression are presented. The left panels show the results of cutoff optimization by maximizing the standardized log-rank statistic. The right panels show the stratification of patients into two strata based on the genes’ optimized z-score cutoff. The drawn *p*-value describes the adjusted *p*-value returned by the maxstat::maxstat.test method. The number of patients at risk at a given time and the cumulative sum of events per stratum were described in the number at risk tables. The inconsistent number of observations between the overall survival (OS) and disease-free survival (DFS) analyses resulted from incomplete DFS data.

Next, we used a univariate Cox proportional hazards regression model to analyze the relationship between AATs expression and survival time, alongside known prognostic factors, including stage, grade, histopathological type, age, and global mRNA and miRNA expression patterns. Some AATs were found to be significant in predicting time-to-recurrence in disease-free survival (DFS) and time-to-death in overall survival (OS) analyses. Specifically, SLC38A3, SFXN4 and SLC3A2 were associated with shorter DFS (*p* =3.67 × 10^−3^; *p* = 0.04, *p* = 0.04, respectively, Figure 5a). Conversely, increased SLC6A6 and SLC1A4 expression levels were associated with longer DFS, with a significantly longer OS for the latter (*p* = 0.03; Figure 5b). Consistent with the link to poorly differentiated, more advanced tumors or high-copy number profile, SLC38A1, SLC38A3, and SFXN1 were related to worse OS (*p* = 0.01; *p* = 3.14 × 10^−3^; *p* = 0.04, respectively; Figure 5b). Importantly, both DFS and OS analyses confirmed well-known prognostic factors, demonstrating that the EC patient dataset is clinically representative and reliable. We further investigated the relationship to prognosis by analyzing the separation of survival curves of stratified patients. By maximizing the log-rank statistic, we identified cutoff values for standardized AAT mRNA expression levels that stratify the population into two groups with significantly distinct prognoses. High levels of SFXN5 were related to unfavorable DFS (*p* = 1.23 × 10^−2^; Figure 6a) contrasting with increased SLC1A4 (*p* = 3.4 × 10^−3^; Figure 6b), SLC6A20 (*p* = 2.91 × 10^−2^; Figure 6c), and SLC6A6 (*p* = 2.3 × 10^−3^; Figure 6d) that identified groups with longer disease-free survival. Again, the glutamine transporters SLC38A1 and SLC38A3, consistent with their association with higher grading, staging, or an abnormal p53 profile, were linked to poor survival (*p* = 2.92 × 10^−2^, Figure 6e and *p* = 1.96 × 10^−2^, Figure 6f).

## 4. Discussion

In this study, we analyzed the expression profiles of 18 AATs in EC and identified several markers that were significantly associated with clinical parameters and had prognostic value. Our results reveal that the expression pattern of AATs identified a patient group with a significantly worse prognosis. We report SLC38A1, SLC38A3, SFXN1, and SLC3A2 as negative prognostic factors, and SLC1A4 as a favorable prognostic marker. By analyzing these data alongside clinical parameters and the TCGA molecular classification, we demonstrate that AAT expression might be linked to tumor aggressiveness and may improve patient stratification. Consequently, it may allow more personalized adjuvant treatment decisions.

EC cells, like other carcinomas, exhibit greater metabolic needs to sustain their accelerated proliferation and survival. This includes an increased demand for essential and nonessential amino acids, resulting in upregulation of specific cell-surface transporters [26,27]. To date, studies have primarily centered on LAT1 (SLC7A5) and xCT (SLC7A11), establishing them as key mediators of nutrient uptake, homeostasis, and chemoresistance in EC, as well as prognostic factors [22,28,29,30,31,32,33]. However, most existing literature has examined these transporters individually, and comprehensive data regarding their prognostic impact remains limited. Our study currently provides a comprehensive analysis of AATs expression in a TCGA-based EC cohort, filling a critical gap in our understanding of how these proteins contribute to disease aggressiveness and poor clinical outcomes.

Among the AATs investigated in our study, both SLC38A1 and SLC38A3 were consistently identified as indicators of poor clinical outcome. Both transporters exhibited an increase in expression corresponding to higher tumor grade and advanced FIGO stage, and to the highly aggressive copy-number-high molecular subtype. Biologically, both proteins function as primary cellular importers of glutamine [24,25]. Glutamine is an essential nutrient for growing malignancies, supporting the tricarboxylic acid cycle and nucleotide biosynthesis. Recent multi-omics analyses have confirmed that glutamine functions as a major energy source for aggressive EC [27]. Furthermore, glutamine metabolism in EC has been shown to facilitate the development of an immune-suppressive tumor microenvironment, which contributes to worse long-term clinical outcomes [34,35]. The upregulation of SLC38A1 and SLC38A3 observed in our high-risk cohort likely reflects this phenomenon. Recent experimental studies support the relevance of these specific transporters in EC growth. Li et al. demonstrated that upregulation of SLC38A1 enhances glutamine metabolism and promotes EC progression [24], while in vitro studies by Md Fuzi et al. confirmed that RNA-interference-mediated knockdown of SLC38A1 significantly inhibits EC cell proliferation [36]. Furthermore, emerging prognostic models utilizing machine learning and glucose metabolism markers have identified SLC38A3 as a key oncogene whose high expression correlates with malignant cellular traits and poor prognosis in EC [37,38]. While previous research often evaluated these SLC38A1/SLC38A3 markers in isolation or in cell models, our TCGA-based analysis assesses these markers on a broader scale. We demonstrate that overexpression of SLC38A1 and SLC38A3 is strongly associated with advanced disease (SLC38A1 only), high-grade histology, and significantly shorter OS and DFS. However, further studies are needed to consider these transporters as promising prognostic markers and therapeutic targets.

Our analysis further identified SFXN1 as a potential unfavorable prognostic factor. The expression of this transporter demonstrated a gradual increase corresponding with progressive tumor grading (from Grade 1 to Grade 3). Biologically, SFXN1 is a mitochondrial protein responsible for serine transport, a process integral to the serine-glycine-one-carbon metabolic process. SFXN1 is essential for tumor growth, providing the one-carbon units required for nucleotide biosynthesis and maintaining cellular redox homeostasis [39,40,41]. The upregulation of SFXN1 observed in our high-grade EC cohort might suggest that aggressive endometrial tumors may exploit these processes to accelerate their growth. While the specific role of SFXN1 in EC has not been previously described, its oncogenic role has been recognized in studies of various malignancies. Experimental analyses have demonstrated that SFXN1 is overexpressed in lung adenocarcinoma, triple-negative breast cancer, and bladder cancer, where it enhances cell proliferation, metastasis, and therapy resistance [42,43,44,45,46]. Specifically, knockdown of SFXN1 in these models inhibits tumor growth. Our TCGA-based results align with these findings, confirming SFXN1 as a major indicator of EC histological aggressiveness and poor survival.

Interestingly, the molecular distribution of SFXN1 expression in our study exhibited a complex pattern. SFXN1 levels were significantly higher in the hypermutated MMRd and POLEmut groups than in the copy-number-low subtype. This phenomenon may be due to the exceptionally high DNA replication demands of MMRd and POLEmut tumors, as confirmed by recent genomic analyses [47,48]. Because the serine-glycine-one-carbon metabolic pathway is the primary source of nucleotides for DNA synthesis, hypermutated ECs may exhibit greater needs for SFXN1-mediated serine transport to sustain their genomic instability and continuous replication. Targeting SFXN1 could present a new treatment opportunity, particularly for high-grade, highly mutated ECs in cases of unresponsiveness to immunotherapy. However, this approach needs to be tested in further mechanistic studies performed on cell lines and tumor organoids.

Our analysis indicated that SLC3A2 increased with tumor grade, in the copy-number-high subtype, and in patients with shorter DFS. SLC3A2 by itself is not an amino acid transporter; however, it is part of heteromeric amino acid transporters, including LAT1 (SLC7A5) and xCT (SLC7A11) [49]. In particular, its association with LAT1 supports the uptake of essential amino acids such as leucine. Its interaction with xCT facilitates cystine uptake and glutathione synthesis, helping cancer cells maintain redox homeostasis [49]. Increased SLC3A2 expression has been reported in several malignancies and has been associated with tumor progression, metastasis, and resistance to therapy [49]. In endometrial cancer, SLC3A2 is overexpressed and associated with malignant characteristics, and experimental modulation of its expression has affected proliferation, invasion, apoptosis, and tumor growth [50]. It was involved in disulfideptosis, a novel cell death mechanism induced by disulfide stress [50]. Thus, SLC3A2 may represent a potential biomarker and therapeutic target in endometrial cancer, although its functional effects may depend on its specific transporter partners.

In contrast, our analysis identified SLC1A4 as a favorable prognostic indicator in EC. Higher SLC1A4 expression was significantly associated with prolonged OS and DFS, and the transporter was upregulated in low-grade and prognostically favorable copy-number-low/endometrioid, MSI, and POLE subtypes. SLC1A4, also known as ASCT1, is an AAT that mediates the uptake of serine, alanine, cysteine, and glutamine (Table 1). To our knowledge, the favorable prognostic role of SLC1A4 in EC has been previously undocumented and contrasts with findings in other malignancies. SLC1A4 has been characterized as an oncogenic marker in hepatocellular carcinoma, non-small cell lung cancer, gastric cancer, and prostate cancer, where its upregulation increases amino acid transport, promotes therapy resistance, and facilitates metastasis [51,52,53,54,55]. The reason for this phenomenon might lie in the unique biology of endometrial tumorigenesis and the concept of metabolic dedifferentiation. Grade 1 ECs are highly differentiated tumors that retain many morphological and physiological features of the normal endometrium [56], as further confirmed by the high proteomic expression of SLC1A4 in healthy endometrial glandular cells, as reported in the Human Protein Atlas [57]. However, as tumors progress to a highly aggressive phenotype, cancer cells appear to downregulate SLC1A4-mediated transport and activate alternative pathways, such as upregulation of SLC38A1, SLC38A3, and SFXN1. Therefore, this downregulation of SLC1A4 could indicate tumor progression and may aid risk stratification.

The integration of these metabolic findings holds significant clinical potential. Despite the expansion of the molecular classification system, predicting patient outcomes and selecting appropriate adjuvant therapies in EC remain clinical challenges. Currently, a proportion of patients are classified as low- or intermediate-risk based on standard clinico-pathological and molecular criteria, yet they unexpectedly experience disease recurrence. Our findings suggest that evaluating upregulation of glutamine and serine transporters, alongside the loss of SLC1A4, could help improve stratification. Consequently, patients who initially appear not to require adjuvant therapy could be accurately identified as high risk, enabling treatment intensification. Furthermore, these specific AATs may be considered as promising therapeutic targets for patients with advanced or chemoresistant EC.

While our study provides insights, several limitations must be acknowledged. Firstly, our analysis is based on retrospective data from the TCGA database; therefore, these data require validation in independent, prospective clinical cohorts. Secondly, our investigation relies on mRNA expression levels, which may not completely reflect the activity of the tumor cell protein due to post-transcriptional and post-translational modifications. Finally, while the literature supports our findings, further studies are necessary to fully explain the molecular mechanisms by which these specific AATs stimulate or inhibit EC progression.

## 5. Conclusions

In conclusion, our analysis identifies mainly SLC38A1, SLC38A3, and SFXN1 as unfavorable prognostic factors, and SLC1A4 as a good prognostic marker in endometrial cancer. The expression of these amino acid transporters correlates with histological grade, disease stage, and the copy-number-high molecular subtype. Integrating these metabolic findings with current molecular classification may improve risk stratification, and these AATs may serve as potential targets for personalized therapies.

## Figures and Tables

**Table 1:** Characteristics, cellular localization, and biological function of the 18 selected AATs.

AAT	Full Name	Cellular Localization	Function	Reference
SFXN1	Sideroflexin-1	Mitochondrion	Transport of serine, alanine, cysteine, cytoplasmic Fe^2+^, and one-carbon metabolism.	[18,19]
SFXN2	Sideroflexin-2	Mitochondrion	Mitochondrial transporter (serine transport, iron homeostasis).	[19]
SFXN3	Sideroflexin-3	Mitochondrion	Mitochondrial transporters, iron homeostasis, mitochondrial respiratory chain regulation, and transport of serine, one-carbon metabolism.	[19]
SFXN4	Sideroflexin-4	Mitochondrion	Mitochondrial transporters, iron homeostasis, and mitochondrial respiratory chain regulation.	[19]
SFXN5	Sideroflexin-5	Mitochondrion	Mitochondrial amino-acid transporter (citrate).	[19]
SLC1A4	Neutral amino acid transporter A	Plasma membrane	Sodium-dependent neutral amino-acid transporter that mediates transport of alanine, serine, cysteine, proline, hydroxyproline, and threonine.	[19]
SLC3A2	Amino acid transporter heavy chain SLC3A2	Plasma membrane	The heavy-chain/chaperone component of heteromeric amino-acid transporters. Transport of multiple amino acids, thyroid hormones, and L-DOPA, and participation in mTORC1 signaling. (As a heterodimer with other SLC7 transporters).	[19]
SLC6A6	Sodium-and chloride-dependent taurine transporter	Plasma membrane	Mediates sodium- and chloride-dependent transport of taurine and beta-alanine, and (possibly) hypotaurine and gamma-aminobutyric acid (GABA).	[19]
SLC6A7	Sodium-dependent proline transporter	Plasma membrane	Transport of proline.	[19]
SLC6A9	Sodium-and chloride-dependent glycine transporter 1	Plasma membrane	Transport of glycine.	[19]
SLC6A20	Sodium-and chloride-dependent transporter XTRP3	Plasma membrane	Transport of glycine, proline, and N-methylated amino acids. SLC6A20 correlates with immune infiltration in endometrial carcinoma.	[19,20]
SLC7A5	Large neutral amino acids transporter small subunit 1	Plasma membrane	Transport of phenylalanine, tyrosine, leucine, histidine, methionine, tryptophan, valine, isoleucine, glutamine, asparagine, alanine, thyroid hormones, and melphalan. Mediates cell growth in EC.	[19,21,22]
SLC15A3	Solute carrier family 15 member 3	Lysosome/Endosome	Transport of histidine, certain di- and tripeptides. including carnosine and mimic peptide drugs—participation in the immune response and the mTOR pathway.	[19,23]
SLC15A4	Solute carrier family 15 member 4	Lysosome/Endosome	Transport of histidine, certain di- and tripeptides. Including carnosine. Participation in the immune response and the mTOR pathway.	[19,23]
SLC25A38	Mitochondrial glycine transporter	Mitochondrion	Mitochondrial glycine transporter that imports glycine into the mitochondrial matrix.	[19]
SLC38A1	Sodium-coupled neutral amino acid symporter 1	Plasma membrane	Transport of glutamine, alanine, asparagine, histidine, serine, cysteine, methionine, glycine, threonine, and proline. Enhancing glutamine metabolism.	[19,24]
SLC38A3	Sodium-coupled neutral amino acid transporter 3	Plasma membrane	Transport of glutamine, asparagine, and histidine.	[19,25]
SLC66A1	Lysosomal amino acid transporter 1 homolog	Lysosome	Lysosomal/vacuolar export of cationic amino acids.	[19]

**Table 2:** Clinicopathological and molecular characteristics of the study cohort. Differences in cohort sizes (*n*) reflect missing clinical data within the TCGA dataset.

Characteristic	Result
**General Characteristics**	(*n* = 373; 100%)
Mean age, years (SD)	63.1 (11.0)
Median age, years (Range)	63 (31–90)
Overall survival data	(*n* = 371; 99.5%)
Death events	39 (10.5%)
Mean OS, years (SD)	2.58 (2.23)
Disease-free survival data	(*n* = 334; 89.5%)
Recurrence events	72 (20.5%)
Mean DFS, years (SD)	2.4 (2.09)
**TCGA Molecular Subtype**	(*n* = 232; 62.2%)
Copy-number-high	60 (25.9%)
Copy-number-low	90 (38.8%)
MMRd	65 (28.0%)
POLE	17 (7.3%)
**Grade**	(*n* = 333; 89.3%)
Grade 1	78 (23.4%)
Grade 2	91 (27.3%)
Grade 3	164 (49.3%)
**FIGO**	(*n* = 370; 99.2%)
Stage I	254 (68.1%)
Stage II	25 (6.7%)
Stage III	74 (19.8%)
Stage IV	17 (4.6%)
**FIGO combined**	(*n* = 370; 99.2%)
Stage I/II	279 (74.8%)
Stage III/IV	91 (24.4%)
**Histological subtype**	(*n* = 373; 100%)
Endometrioid	307 (82.3%)
Mixed	13 (3.5%)
Serous	53 (14.2%)

## Data Availability

Transcriptome data were obtained from TCGA via http://www.cbioportal.org/api/v2/api-docs (accessed on 5 February 2026). We used the clinical characterization of the patients obtained by the function cBioPortalData: clinicalData.
